# Low‐Density Neutrophils: Enigmatic Cells in Health and Disease

**DOI:** 10.1155/jimr/3037744

**Published:** 2025-12-15

**Authors:** Carlos Blanco-Camarillo, Carlos Rosales

**Affiliations:** ^1^ Instituto de Investigaciones Biomédicas, Universidad Nacional Autónoma de México, Mexico City, Mexico, unam.mx; ^2^ Posgrado en Ciencias Biológicas, Universidad Nacional Autónoma de México, Mexico City, Mexico, unam.mx

**Keywords:** cancer, inflammation, NETosis, neutrophil, SLE

## Abstract

Neutrophils have been generally considered to be homogeneous cells with only antimicrobial functions. Nowadays, however, it is clear that neutrophils are heterogeneous cells, with multiple phenotypes and functional states. One neutrophil subpopulation, the low‐density neutrophils (LDN) are found among peripheral blood mononuclear cells (PBMC) after separating blood cells by density gradient centrifugation. LDN have attracted a lot of interest because they increase dramatically in several pathological conditions. LDN have been mostly studied in systemic lupus erythematosus (SLE). In this disease, LDN are prone to produce neutrophil extracellular traps (NETs) and to secrete larger amounts of proinflammatory cytokines. However, in the context of cancer, LDN are described as immunosuppressive cells and have been called granulocytic‐myeloid‐derived suppressor cells (G‐MDSCs). Moreover, in the case of many other diseases, there is very little information on the functional properties of LDN. Hence, LDN not simply increase in numbers but become functionally different during distinct disease states. This has created confusion in the field, and the characteristics and functions of LDN continue to be a contentious issue. In this review, we aim to bring together current research in the field of LDN. We discuss discrepancies in the literature in relation to the identification and functional characterization of LDN, and also the possibility that LDN could become a biomarker for some inflammatory conditions and even novel therapeutics for certain diseases.

## 1. Introduction

Neutrophils comprise about 70% of all leukocytes in human peripheral blood [1]. Neutrophils are regarded as a primary line of defense of innate immunity because they arrive first to tissues with inflammation or infection [2, 3]. Once neutrophils arrive at compromised tissues, they engage in antimicrobial functions such as phagocytosis, degranulation, and generation of neutrophil extracellular traps (NETs) [4–6]. Traditionally, neutrophils have been only considered antimicrobial leukocytes with a short life‐span [7]. However, recently this view has changed. Nowadays, neutrophils are known to participate both in the innate immune response and in the adaptive immune response [8]. In addition, neutrophils have always been assumed to be a homogenous cell population, even though early reports suggested the existence of neutrophil heterogenicity based on differences in migration, phagocytosis, and receptor expression [9]. Today, however, it is clear that neutrophils with multiple phenotypes and functional states are found in conditions such as inflammation, autoimmunity, and cancer [10–13], confirming the functional heterogeneity of neutrophils [14, 15]. One neutrophil subpopulation, the so‐called low‐density neutrophils (LDN), has attracted a lot of interest because LDN increase in several diseases [16–18].

**Figure 1:**
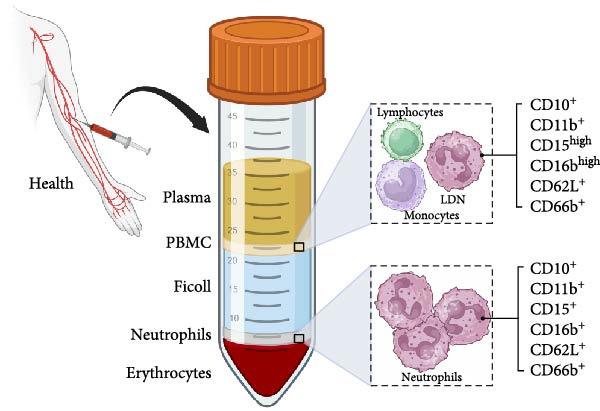
Purification of neutrophils and low‐density neutrophils. Peripheral blood from healthy human donors is placed on top of Ficoll‐Paque and centrifuged. Erythrocytes sediment at the bottom of the tube. Neutrophils are found on top of erythrocytes. Peripheral blood mononuclear cells (PBMC) are found between the plasma and Ficoll‐Paque layers. Neutrophils are mature cells with a segmented nucleus and a normal phenotype (CD10^+^, CD11b^+^, CD15^+^, CD16b^+^, CD62L^+^, and CD66b^+^). PBMC comprise lymphocytes, monocytes, and low‐density neutrophils (LDN), which display a mature nucleus and an activated phenotype (CD10^+^, CD11b^+^, CD15^high^, CD16b^high^, CD62L^+^, and CD66b^+^). Figure created with BioRender.com.

LDN were first reported as neutrophils “contaminating” peripheral blood mononuclear cells (PBMC) in blood from systemic lupus erythematosus (SLE) or rheumatoid arthritis patients [19]. The method for purifying leukocytes involves density gradient centrifugation [20, 21]. First, blood is layered on top of a density medium, and then centrifuged to segregate cells according to their density. Neutrophils appear on top of erythrocytes, separated from PBMC, which for being more buoyant, remain in the low‐density fraction between the density medium and the plasma (shown in Figure 1). For many years, it was believed that the PBMC layer contained only monocytes and lymphocytes. Nowadays, it is clear that a small number of cells among the PBMC from the blood of healthy individuals are neutrophils [22, 23]. These more buoyant neutrophils are the LDN.

LDN have been predominantly studied in the context of SLE [16, 24–26]. However, more recently, LDN have also been reported to be associated with several inflammatory diseases, including juvenile idiopathic arthritis (JIA) [27], pyogenic arthritis, pyoderma gangrenosum and acne (PAPA) syndrome [28], psoriasis [29, 30], asthma [31], sepsis [32–36], and antineutrophil cytoplasmic antibody (ANCA)‐associated vasculitis [37, 38]. LDN are also reported to increase in several infections, including HIV [39, 40], SARS‐CoV‐2 [41–45], *Plasmodium* sp. [46], *Mycobacterium* [47, 48], and *Leishmania* sp. [49]. In addition, LDN are reported to increase in pregnancy [50–53], obesity [54], and cancer [55–58].

LDN have been reported to be proinflammatory in the context of SLE [24, 25, 59]. However, in the context of cancer, LDN are described as immunosuppressive [55, 57]. Therefore, LDN display distinctive functions in different conditions. Moreover, in the case of many other diseases, there is very little information on the functional properties of LDN. In consequence, the characteristics and functions of LDN remain controversial. In this review, we present the current status of research in the field of LDN, particularly during inflammatory and infectious diseases. We discuss discrepancies in the field in relation to the identification and functional characterization of LDN, and also the possibility that LDN could become a biomarker for some inflammatory conditions and even novel therapeutics for certain diseases.

## 2. Origin of LDN

LDN increase in several inflammatory conditions, but it is not clear where and how these cells are generated. Several possibilities proposed in the literature are discussed next.

### 2.1. LDN as Immature Neutrophils Released From the Bone Marrow

Reports on LDN from SLE patients indicated that within these cells, there was dissimilarity in nuclear morphology [24, 60]. Using transmission electron microscopy, it was found that about 40% of cells had an immature nuclear morphology (as shown in Figure 2) [59, 60]. Also, genomic analysis showed that LDN had an expression signature (upregulated granule protein synthesis) typical of early stages of neutrophil development [24, 67]. In addition, neutrophil progenitors from the bone marrow were reported to have a lower density than mature neutrophils [68]. Based on this evidence, it has been proposed that LDN are neutrophil progenitors released from the bone marrow [59]. This idea is supported by the action of some stimuli, such as granulocyte colony‐stimulating factor (G‐CSF) and lipopolysaccharide (LPS), that induce emergency granulopoiesis [69], leading to increased numbers of immature LDN [64, 66, 70]. However, expression of membrane markers indicates that LDN have a mature phenotype (as shown in Figure 2). LDN in SLE are typically positive for CD10, CD15, and CD16 expression [60]. Similarly, LDN from healthy individuals showed a mature phenotype with expression of CD10, CD15, and CD16 [22]. Therefore, LDN seem to be a mixture of cells with both mature and immature phenotypes depending on the disease context.

Figure 2Several possibilities proposed in the literature for the origin of LDN. Low‐density neutrophils (LDN) increase in several pathological conditions, but it is still not clear how LDN are generated. (a) LDN may be immature neutrophils released from the bone marrow [17, 24, 59–61], or LDN may be mature neutrophils with more buoyancy (acquired by unknown mechanisms) [22, 25, 59, 60, 62]. (b) LDN may be activated (primed) neutrophils [22, 59]. (c) LDN may be degranulated neutrophils, which for having less granules become more buoyant [5, 63]. (d) LDN may be intermediate‐mature neutrophils. These cells express at the same time both mature (CD10^+^) and immature (CEBPA and IRF8 transcription factors) cell markers. Thus, they may be cells in the process of a continuous activation state [24–26, 64, 65]. (e) LDN may be a separate neutrophil lineage produced from genomic abnormalities [66]. Arrows indicate higher expression. Figure created with BioRender.com.

**Figure:**
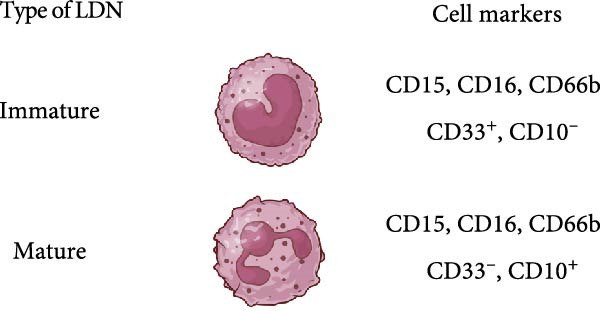
(a)

**Figure:**
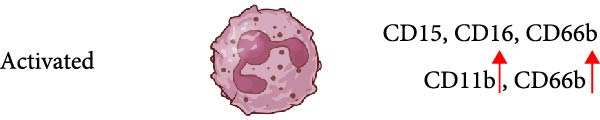
(b)

**Figure:**
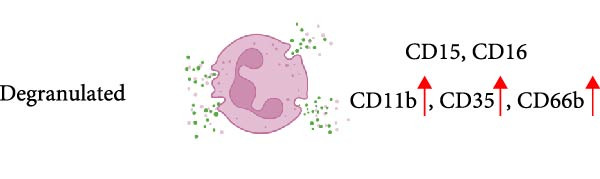
(c)

**Figure:**
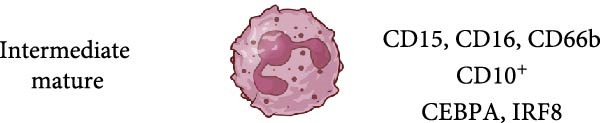
(d)

**Figure:**
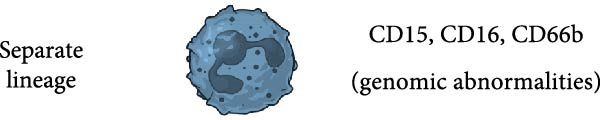
(e)

### 2.2. LDN as Activated (Degranulated) Neutrophils

In addition, LDN in SLE and in healthy individuals exhibit an activated phenotype with higher expression of CD11b, and CD66b [22, 60] (as shown in Figure 2). Therefore, it has also been proposed that LDN arise from the activation of neutrophils. In fact, stimulation of neutrophils in vitro with N‐formylmethionil‐leucyl‐phenylalanine (fMLF) [64], with interferon (IFN)‐*α* [71], or with ascites fluid from patients with ovarian cancer [72], led to an increase in LDN. Also, these LDN consistently expressed higher levels of activation molecules (CD11b, CD16, CD35, and CD66b) [64]. A conceivable mechanism for this neutrophil change in buoyancy is degranulation [5, 63] (as shown in Figure 2). Nevertheless, electron microscopy analysis of LDN in SLE did not show evidence of degranulation [59], and in patients with sporotrichosis, a fungal infection, LDN did not exhibit significant changes in degranulation either [73]. From the previous discussion, it is clear that after degranulation, some neutrophils could become LDN, but degranulation alone does not fully explain the formation of all LDN.

### 2.3. LDN as Neutrophils With a Continuous Spectrum of Activation

Based on transcriptomic and genomic analysis, LDN from SLE patients are described as subpopulations with various degrees of activation and intermediate maturation states. LDN display different degrees of chromatin accessibility at sites of multiple transcription factor motifs [24–26]. The CD10^+^ subset, for example, is considered “mature” cells. However, these cells also express transcription factors (CEBPA and IRF8) normally associated with immature neutrophils [65]. Hence, these LDN were reported as intermediate‐mature cells [24] (as shown in Figure 2). Consequently, LDN subsets may be just activated neutrophils with a continuous spectrum of activation [64]. At present, we have almost no information on what signals could induce the appearance of LDN. In a recent study, in vitro infection of neutrophils with *Mycobacterium tuberculosis* led to the development of LDN by a process requiring NET formation and reactive oxygen species (ROS) [74]. In other conditions, there are no reports on what factors may induce LDN.

### 2.4. LDN as a Separate Neutrophil Lineage

Opposite to the cell activation origin of LDN, it is also suggested that LDN originate from a separate neutrophil lineage resulting from genomic abnormalities, such as an elevated copy number of genes [66] (as shown in Figure 2). Consequently, it remains unclear whether LDN are an abnormally developed neutrophil subset distinct from normal neutrophils, or more likely a continuous spectrum of activated neutrophils in response to disease‐specific inflammatory cues.

## 3. Characterization of LDN

An obligated condition for neutrophils to be called LDN is to be more buoyant in density gradients. Therefore, the first step in characterizing LDN must always involve centrifugation on a density gradient. Next, identification and classification of LDN among the PBMC is usually achieved by detecting the expression of cell membrane molecules via flow cytometry.

However, the description of cells based on their membrane phenotype is complex due to cell heterogeneity and to a lack of consensus on what molecules are relevant to examine. In the general absence of CD14, a monocyte marker [75], and the presence of CD15, a granulocyte marker [76], are used to identify LDN within the PBMC. So, LDN have been defined as CD14^−^, CD15^+^ cells in SLE [59], and in other inflammatory conditions [28, 77–79] (Table 1). Nevertheless, because CD15 is not only expressed on neutrophils, some investigators have used the term low‐density granulocytes (LDGs) when referring to these proinflammatory cells (Table 1) [24, 80]. The term LDG, however, suggests that eosinophils and basophils may also be present [61]. But the presence of eosinophils has not been examined in most reports. In a study of patients with chronic kidney disease, no expression of siglec‐8, an eosinophil‐specific marker [91], was detected amid CD15^+^ cells [92], suggesting that no eosinophils were present among LDN. In accordance with this result, eosinophils are known to express much lower levels of CD15 than neutrophils [93]. Therefore, since most studies select cells based on CD15 expression, it is most likely that these leukocytes are mainly neutrophils. In fact, in SLE only LDN, and no eosinophils, have been described [60, 94]. Thus, we think the term LDN is more adequate while referring to these cells. Still, there is no agreement on this issue, and some maintain that the terms LDG and LDN can be used interchangeably [44, 95].

**Table 1:** Different names given to low‐density neutrophils.

Cell name	Phenotype	Health condition	Main function	Reference
LDN	CD10^+^, CD14^–^, CD15^+^, CD16b^+^, CD66b^+^	Health	?	[22, 23]
LDN	CD14^–^, CD15^+^, CD16^+^	SLE	Proinflammatory	[60, 61]
		AAV		[79]
	CD14^–^, CD15^+^	Tuberculosis		[47]
		COVID‐19		[45]
LDG	CD14^lo^, CD15^+^, CD10^+^	SLE,	Proinflammatory	[24, 25, 80]
		Myasthenia gravis		[81]
		Asthma		[82]
		Pregnancy		[83]
MDSC	CD11b^+^, CD15^+^ (mixture of granulocytic CD14^–^and monocytic CD14^+^ cells)	Cancer	Immunosuppresion	[57, 84, 85]
G‐MDSC	CD11b^+^, CD15^+^, CD14^–^	Cancer	Immunosuppresion	[62, 63, 86–90]
M‐MDSC	CD14^+^ (monocytes)	Cancer	Immunosuppresion	[57]

Abbreviations: AAV, antineutrophil cytoplasmic antibody (ANCA)‐associated vasculitis; COVID‐19, coronavirus disease 2019; G‐MDSC, granulocytic myeloid‐derived suppressor cells; LDG, low‐density granulocytes; LDN, low‐density neutrophils; MDSC, myeloid‐derived suppressor cells; M‐MDSC, monocytic myeloid‐derived suppressor cells; SLE, systemic lupus erythematosus.

Other membrane molecules associated with neutrophils, such as CD11b, CD16, CD66b, and CD10, as well as not associated with neutrophils, such as CD64, and HLA‐DR, have also been used for distinguishing LDN. Table 2 shows the combination of membrane markers used in several reports identifying LDN in multiple pathologies. A more complete phenotype for LDN as defined in SLE is CD3^−^, CD19^−^, CD20^−^, CD56^−^, and CD11b^+/high^, CD14^−/low^, CD15^+^, CD16^+^, CD33^+^, and CD66b^+/high^ [61]. In addition, based on their CD10 expression, these LDN have been described as mature CD10^+^ LDN or immature CD10^−^ LDN [17, 61] (as shown in Figure 2). Interestingly, LDN from healthy individuals were characterized as CD10^+^, CD11b^+^, CD14^−/low^, CD15^+/high^, CD16b^+/high^, CD62L^+^, CD66b^+^, and CXCR4^+^ [22], indicating they are mature cells. These healthy LDN, similarly to LDN in SLE [59], also display an activated phenotype. The molecule CD33 has also been used in several studies to describe mature (CD33^−^) from immature (CD33^+^) cells [25, 62, 99] (as shown in Figure 2).

**Table 2:** Membrane protein expression (phenotype) for selection of low‐density neutrophils.

Condition	Phenotype	Cell name	Reference
Health	CD10^+^, CD11b^+^, CD14^–/low^, CD15^+/high^, CD16b^+/high^, CD62L^+^, CD66b^+^, CXCR4^+^	LDN	[22]
SLE	CD14^low^, CD15^+^, CD10^+^	LDN	[60]
SLE	CD3^–^, CD19^–^, CD20^–^, CD56^–^, and CD11b^+/high^, CD14^–/low^, CD15^+^, CD16^+^, CD33^+^, and CD66b^+/high^	LDN	[61]
SLE	CD14^–/low^, CD15^+^		[26]
SLE	CD11b^+^, CD33^+^, CD15^+^	LDG	[25]
Cancer			
Cancer	CD11b^+^, CD14^–^, CD15^+^ or CD66b^+^ CD11b^+^, CD14^+^, HLA‐DR^–/lo^, CD15^–^	G‐MDSC M‐MDSC	[86]
Advanced cancer	CD33^+^, CD11^+^, CD14^–/low^, CD15^+/bright^	G‐MDSC	[88]
Breast cancer	CD11b^+^, CD14^–^, CD15^+^, CD66b^+^	G‐MDSC	[96]
Hodgkin and non‐Hodgkin lymphoma	CD66b^+^, CD33^dim^, HLA‐DR^–^	G‐MDSC	[62]
Non‐small cell lung cancer	HLA‐DR^–/low^, CD14^–^, CD11b^+^, CD15^+^	G‐MDSC	[90]
Cancer	CD15^+^		[87]
Renal cell carcinoma	CD14^–^, CD11b^+^, CD66b^+^	MDSC	[63]
Multiple myeloma	CD11b^+^, CD14^–^, CD15^+^, CD33^+^, HLA‐DR^–^	G‐MDSC	[89]
Autoimmunity			
Multiple sclerosis	CD14^+^, HLA‐DR^low^ CD33^+^, CD15^+^, CD11b^+^, HLA‐DR^low^	M‐MDSCs G‐MDSC	[97]
Myasthenia gravis	???	LDG	[81]
AAV	CD14^–/lo^, CD15^+^, CD10^+^	LDN	[38]
AAV	CD14^–^, CD15^+^, CD16^+/int/hi^	LDN	[79]
AAV	CD14^lo/int^, CD15^+/hi^, CD66b^+^, CD10^+/hi/lo^	LDN	[37]
Rheumatoid arthritis	CD14^+^, CD15^+/hi^, CD16^–/lo^		[19, 98]
PAPA syndrome	CD14^–^, CD15	LDN	[28]
Myocardial Infarction	CD3^–^, CD19^–^, CD14^–^, CD15^+^, CD66b^+^, CD33^high^, CD16^low^ CD33^low^, CD16^high^	LDN Neutrophils	[99]
Inflammation			
Psoriasis	CD14^lo^, CD15^hi^, CD10^hi^	LDN	[30]
Psoriasis	CD14^–^, CD16^+^, CD66^+^	LDN	[100]
GVHD	CD33^+^/^high^, CD66b^+^/^high^, HLA–DR^low^, IL‐4R*α* ^+^, CXCR4^+^	G‐MDSC	[101]
Chronic GVHD	CD14^–^, CD66b^+^	LDN	[102]
Asthma	??	LDG	[31]
Surgery	CD66b^+^	LDN	[82]
Surgery	CD66b^+^	LDN	[103, 104]
Trauma	CD15^+^, CD66b^+^	LDN	[105]
Infections			
Sepsis	CD14^–^, CD15^+^	G‐MDSC	[36]
Gram‐positive sepsis	CD14^–^, CD11b^+^, CD15^+^, CD33^+^	G‐MDSC	[33]
Tuberculosis	CD14^–^, CD15^+^	LDN	[47, 48]
Sporotrichosis	CD16^+^, CD66b^+^	LDN	[73]
Malaria	CD14^–^, HLA‐DR^–^, CD66b^+^, CD15^+^, CD16^+^	LDN	[46]
Visceral leishmaniasis	CD15^+^, Arginase^+^		[49]
HIV	CD3^–^, CD14^–^, CD15^+^, Arginase^+^	LDN	[39]
COVID‐19	CD11b^+^, CD14^–^, CD15^+^, CD33^+^, CD66b^+^, HLA‐DR^–/low^	LDN = G‐MDSC	[44]
COVID‐19	CD3^–^, CD56^–^, CD19^–^, CD14^–^, CD66^+^, CD15^+^	LDN	[41]
COVID‐19	CD14^–^, CD15^+^	LDN	[45]
Pregnancy			
Pregnancy	CD3^–^, CD14^–^, CD15^+^, Arginase^+^	LDN	[106]
Pregnancy	CD14^–^, CD15^+^	LDG	[83]
Pregnancy	CD66b^+^	G‐MDSC	[107]
Neonate (cord blood of preterm infants)	CD14^–^, HLA‐DR^–/low^, CD66b^+^, CD33^+^	G‐MDSC	[108]
Neonate	CD14^–^, CD11b^+^, CD15^+^	G‐MDSC	[51]
Neonate	CD66b^+/hi^, CD33^+/hi^, IL‐4RA^/inter^, HLA‐DR^–^	G‐MDSC	[109]
Neonate	CD66b^+^	G‐MDSC	[110, 111]

Abbreviations: AAV, antineutrophil cytoplasmic antibody (ANCA)‐associated vasculitis; COVID‐19, coronavirus disease 2019; G‐MDSC, granulocytic MDSC; GVHD, graft‐versus‐host disease; HIV, human immunodeficiency virus; LDN, low‐density neutrophils; M‐MDSCs, monocytic MDSCs; MDSC, myeloid‐derived suppressor cells; PAPA, pyogenic arthritis, pyoderma gangrenosum and acne; SLE, systemic lupus erythematosus.

A serious drawback in characterizing these cells only by flow cytometry is the tendency to assign a particular cell type or function to a certain phenotype. For example, in some studies on myocardial infarction, LDN were defined as CD33^high^, CD16^low^ cells, while neutrophils were defined as CD33^low^, CD16^high^ cells [99, 112]. This simple approach would select preferentially immature cells while ignoring mature LDN. And, as we have mentioned, LDN are composed of a mixture of mature and immature cells in SLE. Thus, LDN cannot be selected only on the basis of CD33 expression. Similarly, in a report on periodontitis, LDN subsets were defined as “normal” (CD16^bright^, CD62L^bright^), “bands” (CD16^dim^, CD62L^bright^), “suppressive” (CD16^bright^, CD62L^dim^), and “active” (CD16^bright^, CD62L^negative^) [113]. These subsets were not confirmed by microscopic or functional studies. Because, there is not any report indicating that a certain phenotype corresponds to a specific cellular morphology or function, this practice should be discouraged. Adding to this complex scenario is the fact that there is not a consensus on what a meaningful level of expression is for any particular molecule. Thus, expression levels are reported not only as “positive or negative," but also as “bright/high or dim/low.” Since different antibodies, fluorochromes, and instruments with various sensitivity levels are used, there is a growing feeling that common criteria for a consistent terminology are required [86, 114, 115].

In the meantime, we put forward a few recommendations that should help in interpreting results from different reports. In all cases, LDN should always be studied within PBMC in order to guarantee they are low‐density cells. For flow cytometry analysis, a detailed description (including catalog number, brand, and concentration) of antibodies and fluorochromes used is required. Also, the gating strategy must always be included. A phenotype described by flow cytometry analysis is not enough to establish a particular activation state or cellular function. After a certain cellular subset is identified, the cells must be purified either by cell sorting or with magnetic beads to perform functional studies. Only after a cellular function is confirmed in cells with a particular phenotype, this association can be established.

Another important issue on the detection and characterization of LDN is the time between blood collection and separation of cells. It is well‐known that neutrophils have a short lifespan, and they cannot be frozen for preservation [116]. Hence, it has been observed that isolating of LDN from frozen PBMC results in significant reduced LDN viability [63]. Therefore, the practice of freezing PBMC for later isolation of LDN, as done in some studies, should be avoided. These different approaches for describing LDN create confusion in the field. Consequently, it would be beneficial to try standardizing the characterization protocols for LDN studies.

## 4. LDN in Health

LDN reported in healthy humans are found with a frequency of around 5% among PBMC [22, 25, 60, 70]. These healthy LDN exhibit typical mature neutrophil morphology and express a CD10^+^, CD11b^+^, CD14^−^, CD15^+/high^, CD16b^+/high^, CD62L^+^, CD66b^+^, and CXCR4^+^ phenotype (Table 2). Also, healthy LDN produced more ROS and phagocytosed better than neutrophils, but they formed NET similarly to neutrophils [22]. Therefore, healthy LDN have a similar morphology to mature neutrophils, but higher expression of activation markers [22, 23, 30, 39, 106], suggesting a primed phenotype. However, currently, there are not any reports indicating what the functions of these healthy LDN might be.

## 5. LDN in SLE

**Figure 3:**
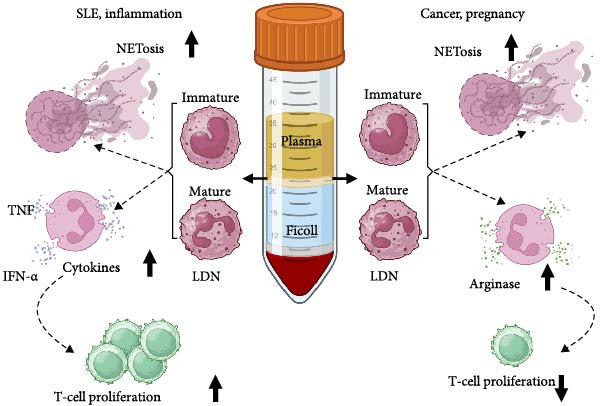
Biologic properties of low‐density neutrophils (LDN) in disease. LDN in pathological conditions are reported to be a heterogeneous population with mature (segmented nuclei) and immature (band) cells. In systemic lupus erythematosus (SLE) and inflammation (left side), LDN display enhanced NETosis, increased production of proinflammatory cytokines, such as tumor necrosis factor (TNF) and interferon alpha (IFN‐*α*), and immune‐promoting activity by inducing T‐cell proliferation. In cancer and pregnancy (right side), LDN display enhanced NETosis, increased production of arginase, and immunosuppressive activity by inhibiting T‐cell proliferation. Figure created with BioRender.com.

LDN are generally associated with SLE since they were first described among PBMC from the blood of SLE patients [19]. Years later, expression of neutrophil‐specific genes, including granulopoiesis‐related and IFN‐induced genes, was found among PBMC from pediatric SLE patients [117]. These genes defined a “granulocyte signature,” which confirmed the presence of LDN within the PBMC layer of SLE patients [117]. In SLE patients with elevated numbers of LDN, many clinical symptoms are exacerbated, including vasculitis [60] and coronary atherosclerosis [24, 118]. Thus, LDN seem to be responsible for many inflammatory complications in SLE. In this disease, LDN consist of a heterogeneous population of CD10^−^ immature neutrophils and CD10^+^ mature neutrophils (as shown in Figure 2). These LDN have reduced phagocytosis [60], display enhanced NETosis [26, 59, 119], secrete increased amounts of proinflammatory cytokines [25, 26, 60], and stimulate T cells to proliferate and produce more proinflammatory cytokines [25]. Hence, SLE LDN display an activated phenotype with proinflammatory properties (as shown in Figure 3) that contribute to a more severe disease [16, 17, 61].

## 6. LDN in Cancer

LDN have also been found to increase in number in individuals with cancer, and they seem to correlate with disease severity [120, 121]. But in most reports, LDN are described as granulocytic‐myeloid‐derived suppressor cells (G‐MDSCs) [62] (Table 1). However, there is great morphological, phenotypic, and functional heterogeneity among MDSC [122], resulting in uncertainty as to whether G‐MDSC from cancer patients are comparable to LDN in other inflammatory conditions. Literature on MDSC in cancer is extensive and beyond the scope of this review. Excellent reviews on MDSC have recently been published [57, 84]. Nevertheless, few comments on the relationship of G‐MDSC in cancer and LDN are granted.

MDSC were firstly identified in the spleens of tumor‐bearing mice and described as immature myeloid cells with suppressive activity over T‐cell responses [123, 124]. MDSC were defined by the expression of the molecules Gr‐1 and CD11b. Later, as research advanced, it became evident that MDSC consisted of not only immature cells but also mature cells, and in addition, they comprised polymorphonuclear neutrophils (CD14^−^) and monocytes (CD14^+^) with distinct immunosuppressive features [94, 125]. The term MDSC was proposed in 2007 to emphasize the heterogeneous nature of these cells [85].

As humans do not express the molecule Gr‐1, MDSC in cancer patients were detected among the PBMC. Human MDSC were then defined as CD11b^+^, CD15^+^ cells with or without CD14 expression (Table 1). Thus, the cells selected, in most studies, as CD11b^+^, CD14^−^, CD15^+^ (and sometimes as CD66b^+^, and CD16^+^) (as shown in Table 2) are indeed LDN. However, these LDN are called G‐MDSC in cancer studies, and their low‐density nature is rarely mentioned [122]. Among the PBMC of cancer patients, another group of cells, positive for CD14 expression, has been defined as monocytic‐MDSC (M‐MDSC) [57] (Table 1). These cells are, in general, not studied in detail, since most reports concentrate in the CD14^−^, CD15^+^ (neutrophil) G‐MDSC population. The name G‐MDSC is also confusing because it suggests that several granulocytes, including neutrophils, eosinophils, and basophils, may be part of the G‐MDSC. However, only neutrophils have been described as part of G‐MDSC [126, 127]. Therefore, in cancer as well as in SLE and other inflammatory conditions, neutrophils found in the PBMC layer are in fact LDN.

These LDN may vary in the expression pattern of membrane molecules and may have different functions depending on the inflammation context they experience [60, 70, 128].

The main characteristic of MDSC in cancer is the suppression of T cells [84]. Unfortunately, in the majority of cancer studies, the actual immunosuppressive activity of the cells is not assessed. Cells are described based only on their membrane protein expression. As we mentioned before, no specific neutrophil function can be assigned to a particular phenotype. So, we find reports describing immunosuppressive activity of G‐MDSC in the PBMC fraction of cancer patients [63, 87]; and we also find reports indicating that G‐MDSC from lung or prostate cancer patients do not inhibit T‐cell responses [88]. Remarkably, in this study, the M‐MDSC were found to suppress T‐cell proliferation [88]. This agrees with a recent meta‐analysis of many reports on patients with solid tumors, which indicates the M‐MDSC subpopulation seems to be a consistent and better prognostic marker for cancer than G‐MDSC [129].

In an effort to find a molecular marker for suppressor cells, the molecule LOX‐1 (lectin‐type oxidized LDL receptor 1) was found to be overexpressed in G‐MDSC and, therefore, proposed to be a marker for immunosuppressive cells [130]. Supporting this idea, a microscopy study on human head and neck cancer tissue reported colocalization of T cells with low expression of the proliferation marker Ki67, and neutrophils (called G‐MDSC) expressing LOX‐1 [131]. This was interpreted as evidence that LOX‐1‐positive cells were immunosuppressive. However, in this study, cells were not isolated, and their actual suppressive activity was not evaluated. Moreover, other reports indicate that cells expressing LOX‐1 were not able to inhibit T‐cell proliferation [25, 102]. Clearly, LOX‐1 is not a marker for immunosuppressive activity. Additionally, MDSCs are, in general, described as immature cells [132]. However, a study examining many reports on immunosuppressive G‐MDSC in human cancers noticed that in the majority of reports (over 85%), the cells had a segmented nucleus, indicating they are indeed mature LDN [126]. Moreover, a recent study on patients with lung or ovarian cancer found that LDN with a mature phenotype also suppressed T‐cell proliferation [133]. Therefore, it is important to emphasize that at present, neither maturity (nuclear morphology) nor phenotype (a single molecule or combination of membrane molecules) can reliably distinguish suppressive cells (LDN or G‐MDSC) in either humans or mice. Consequently, when studying MDSC (either neutrophils or monocytes), it is important to clearly describe the cell population involved, and in all cases, confirm their immunosuppressive activity, before they can be called “suppressor cells.”

Because recently it has become evident that neutrophils are cells with great plasticity [10, 14, 15, 134], the existence of LDN (or G‐MDSC) with several phenotypes, reflects differences in function, and clinical condition of these cells. LDN may display proinflammatory functions in diseases such as SLE, and immunosuppressive activity in cancer (as shown in Figure 3). The different LDN functions may also be the result of the tissue context they experience. This concept has been shown to be the case for neutrophils, which display different functions in diverse healthy tissue environments [10, 135] or in various tumors [136].

## 7. LDN in Other Inflammatory Diseases

In addition to SLE and cancer, LDN numbers have been reported to increase in several other inflammatory diseases. In most reports, however, there is simply a description of the presence of LDN among the PBMC of patients with the disease studied. Further characterization of LDN functional properties has only been briefly investigated in a few diseases. Reports of LDN in some immune‐mediated and other inflammatory diseases will be described next.

### 7.1. Autoimmunity (Table 2)

Multiple sclerosis is a chronic autoimmune disease in which dysregulated T cells induce inflammation of the brain. In patients with relapsing‐remitting multiple sclerosis, an increased amount of LDN (CD11b^+^, CD15^+^, CD33^+^, HLA‐DR^−/low^) as well as M‐MDSC (CD14^+^, HLA‐DR^−/low^) was found during relapse. In this case, LDN did not suppress T‐cell responses [97]. In another report, LDN were also increased in patients with multiple sclerosis and in patients with NMOSD (neuromyelitis optica spectrum disorder). In this report, however, the T‐cell suppressive activity of LDN was not evaluated [137].

Myasthenia gravis is an autoimmune neuromuscular disease. Patients with this disease were found to have larger numbers of LDN, which correlated with disease severity [81]. LDN had enhanced spontaneous NET formation and increased ROS production. But they did not inhibit T‐cell responses. Instead, LDN triggered a proinflammatory Th1/Th17 response. Also, single‐cell analysis reinforced that LDN in myasthenia gravis corresponds to a distinct subtype of neutrophils with proinflammatory functions [81]. In patients with autoimmune hepatitis, a systemic inflammation is frequently observed. In these patients, also an increased number of LDN has been reported. These LDN also expressed more myeloperoxidase (MPO) than neutrophils [138]. Thus, it was suggested that LDN may be a biomarker for increased systemic inflammation and for liver fibrosis. No other functional properties of these LDN were reported.

ANCA‐associated vasculitis (AAV) is an autoimmune disorder where autoantibodies against neutrophil proteins, particularly MPO and proteinase 3 (PR3), are frequently present. Immune complexes with these autoantibodies lead to blood vessel damage. Gene expression analysis of cells in blood of patients with AAV revealed a neutrophil gene signature (including MPO and PR3), which was associated with disease and disappeared in response to treatment. This neutrophil signature was confirmed among PBMC, suggesting the existence of LDN associated with the disease [38]. LDN were confirmed later, comprising a three‐fold increase in active AAV patients over patients in remission and over healthy control individuals [79]. These LDN are heterogeneous, with about 75% mature cells and 25% immature cells, and did not respond to anti‐MPO antibody stimulation. Based on this, it was proposed that LDN were more likely the result of generic emergency granulopoiesis rather than to be a primary driver of AAV pathogenesis [79]. However, LDN from AAV patients were found to produce more NET [37], which correlated with disease severity [139]; and the proportion of mature LDN (CD10^+^) decreased in patients during treatment [37]. Therefore, NET produced by LDN might be a relevant therapeutic target in AAV.

Rheumatoid arthritis is a chronic autoimmune disease that predominantly affects joints by causing inflammation. LDN were originally also found in patients with this disease [19]. Transcriptomic analysis of LDN from rheumatoid arthritis patients revealed that these cells have many (more than 5000) genes differentially expressed from arthritis neutrophils or from healthy neutrophils [98]. Particularly, higher expression of elastase and MPO, and some cell‐cycle genes, including cyclin‐dependent kinases, and lower expression of genes for cytokines and cytokine receptors were observed. These LDN seem to be less functional than neutrophils, since they produced less ROS and less NET. Also, LDN were deficient in chemotaxis and phagocytosis [98]. The possible consequences of these less functional LDN on the disease remain to be elucidated.

JIA is another autoimmune, inflammatory disease that normally affects children under 16 years of age. LDN were also elevated in JIA patients, and their transcriptomic profile showed higher expression of MMP8 (matrix metalloproteinase 8; also known as neutrophil collagenase), which may be implicated in disease pathology through breaking down collagen, causing systemic inflammation and damage to joints [27].

Another form of arthritis is the PAPA syndrome. In this disease, patients have flares of sterile arthritis, characterized by a rich neutrophil infiltrate and overproduction of interleukin (IL)‐1*β*. In PAPA syndrome patients, LDN were also found in larger numbers. These LDN had enhanced NET formation [28]. Also, sera from PAPA patients exhibit impaired NET degradation. Therefore, LDN producing more NET together with a deficient degradation may result in the accumulation of NET, which then promotes further inflammation and aggravate disease [28].

### 7.2. Inflammation (Table 2)

Psoriasis is a chronic inflammatory disease that can involve the skin, joints, or both. Patients with psoriasis are also predisposed to early‐onset atherosclerosis, which may lead to cardiovascular complications. In psoriatic skin lesions, neutrophils are abundantly present and are associated with the pathophysiology of the disease [140, 141]. However, only recently the role of LDN in psoriasis has been investigated. Through gene expression profiling of PBMC from patients with generalized pustular psoriasis (GPP), it was discovered that genes related to neutrophil functions were significantly upregulated, indicating the presence of LDN [142]. The larger numbers of LDN were positively associated with psoriasis severity [30], and these LDN showed increased NET formation capacity. NETs participate in the progress of psoriasis. Diminishing NET formation with DNase I or CI‐amidine in vivo, the severity of the disease is reduced in an imiquimod‐induced psoriasis‐like mouse model [143]. For NET formation, the activity of the enzyme elastase is required. Consequently, psoriasis LDN expressed more elastase and less secretory leukocyte protease inhibitor (SLPI), an elastase inhibitor, than neutrophils [144]. Additionally, platelets have been reported to stimulate neutrophils to release NET [145], and platelets adhered only to LDN but not to neutrophils [30]. Likewise, the numbers of LDN aggregating with platelets were positively associated with early noncalcified coronary plaque burden [30], suggesting a role of NET on cardiovascular complications in psoriasis. In addition, LDN could release IL‐17 via NET formation in psoriasis [29], and NET, with the assistance of monocytes in vitro, could also induce Th17 cells from human PBMC [146]. Together, these reports support a central role for LDN, via NET formation and IL‐17, in the proinflammatory mechanism in psoriasis.

Graft‐versus‐host disease (GVHD) is an immune reaction of cells from transplanted tissue against the host cells. Depending on the onset time and the severity of symptoms, GVHD can be classified as acute GVHD (aGVHD) or chronic GVHD (cGVHD). LDN were found in patients suffering aGVHD after extracorporeal photopheresis treatment. These LDN (CD66b^+^ cells; called G‐MDSC) were reported to suppress T‐cell responses [101] and were interpreted as a good response after treatment to dampen T‐cell functions. However, more recently, in patients with cGVHD, LDN were elevated and reported to be proinflammatory [102]. These LDN (CD66b^+^ cells) were predominantly immature CD10^−^ cells that promoted T‐cell proliferation, and production of IFN‐*γ* and IL‐6 [102]. A possible reason for the discrepancy between the two reports is the higher proportion of immature CD10^−^ LDN, which have been previously reported to enhance T‐cell proliferation [70]. Therefore, LDN may have a pathogenic potential in cGVHD [102], but further research is required to clarify the role of LDN in GVHD.

Asthma is a lung disease involving chronic inflammation of airways that results in bronchoconstriction, mucus production, and difficulty to breathe. Important immunohistopathologic features of asthma include sputum and inflammatory cell infiltration, mainly neutrophils, in the airways. In asthma patients, LDN were also found to be elevated, comprising up to 39% of PBMC, and their numbers were associated with asthma severity [31]. No other function of these cells was reported, but it was proposed that LDN may provide a biomarker for severe asthma.

Surgery is a procedure that can induce strong inflammation. After abdominal surgery, several types of immune cells are detected in the peritoneal cavity. Hence, the role of LDN after surgery in cancer patients has been recently explored. Peritoneal lavages were collected from gastric cancer patients before and after abdominal surgery. The number of LDN (CD66b^+^ cells) was increased in postoperative lavages [103]. Similarly, the number of LDN in peripheral blood was significantly increased [104]. The number of LDN exhibited a positive correlation with surgical time and with the amount of blood lost. These LDN were also immunosuppressive [104]. Purified LDN released NET during a short‐term culture in vitro. These NETs are also efficiently attached to various human gastric cancer cells [103, 104]. Moreover, transfer of purified LDN and gastric cancer cells into nude mice significantly increased metastasis in the peritoneal cavity. This metastasis was inhibited by intraperitoneal administration of DNase I [103]. Similarly, in a study of patients with colorectal cancer who underwent radical surgery, the number of LDN was increased immediately after surgery [82]. Purified LDN produced high amounts of NET, which also efficiently trapped tumor cells in vitro [82]. LDN numbers also correlated positively with surgical time, intraoperative blood loss, and decreased recurrence‐free survival [82]. Together, these reports indicate that LDN are increased after surgical stress, and they can produce more NET, which, by trapping cancer cells, may promote metastasis. So, disruption of NET with DNase might be a potential way to prevent recurrence after surgery.

Another condition that can trigger a systemic inflammatory response is severe injury. In response to tissue trauma, neutrophils get hyper‐activated in peripheral blood, leading to inflammatory complications, including increased susceptibility to infection and even death [147, 148]. To explore how trauma could change neutrophil heterogeneity, a recent study compared neutrophils and LDN from healthy individuals and from trauma patients. Mass‐spectrometry‐based cytometry was used to classify cells according to a panel of 44 distinct protein markers. Several discrete neutrophil populations were determined based on protein membrane expression [105]. Among PBMC from trauma patients, a larger percentage of LDN was found. These LDN seem to be a unique cell population with a phenotype of functional exhaustion, and upregulated expression of active adhesion molecules, particularly the integrin Mac‐1 (CD11b/CD18) [105]. This study provides evidence that circulating neutrophils display functional heterogeneity and that trauma promotes the appearance of a novel, exhausted, LDN population.

### 7.3. Infections (Table 2)

When bacterial infections are severe, they can induce sepsis, which is a serious medical problem leading to organ malfunction. Because neutrophils are central cells for controlling infections, there is a growing interest on discovering possible different neutrophil phenotypes associated with sepsis. Already, an early report indicated the presence of LDN in patients with severe bacterial infections. These LDN had lower chemotactic and beta‐glucuronidase activities [34]. More recently, it was also found that LDN, which expressed arginase and suppressed T‐cell proliferation, appeared in sepsis patients [32, 33, 35, 36]. These LDN were found preferentially in Gram‐positive cases [33]. Based on phenotype and morphological characteristics, LDN in sepsis patients seem to be formed by a mixture of neutrophils at various maturation stages. Also, the phagocytotic capacity and the chemotactic ability of LDN were significantly reduced [35]. A transcriptomic analysis of peripheral blood performed on patients with sepsis and healthy donors confirmed that LDN are specifically expanded in patients with sepsis [36]. Elevated initial numbers of these LDN and arginase levels were also linked to later nosocomial infections [36]. Eliminating LDN (CD14^−^, CD15^+^ cells; called G‐MDSC) from patients with sepsis increased T‐cell proliferation in in vitro assays [36]. A very recent meta‐analysis of gene expression datasets of PBMC from patients with viral and bacterial infections confirmed the presence of LDN in infected patients through the identification of 49 differentially expressed genes commonly associated to neutrophil granule proteins and to neutrophil degranulation pathway [128]. In RNA‐seq dataset analysis, 24 genes were consistently upregulated in severe infections. Several (17) of these genes were also overexpressed in CD16^+^ cells [128]. Similarly, analysis of a proteomics dataset of PBMC from sepsis patients confirmed elevated protein expression in CD16^+^ cells, which was associated with sepsis and septic shock [128]. Together, these reports confirm that severe bacterial infections lead to an increase in LDN, which is associate with disease severity. Therefore, elevated numbers of LDN might be a good biomarker for complications in bacterial infections.

Infections with *Mycobacterium tuberculosis* [47] or *Mycobacterium leprae* [48] also cause an increase in LDN. These LDN had a segmented nuclear morphology, indicating mature cells that also displayed an activated phenotype [47, 48]. Also, in sporotrichosis, a subcutaneous fungal infection, LDN were significantly elevated and positively correlated with disease severity [73]. Similarly, in infections with parasites, LDN have been reported to be increased. In malaria patients, infected with *Plasmodium* sp., LDN were found [46], and in patients with visceral leishmaniasis, infected with *Leishmania* sp., arginase‐expressing LDN were also found [49]. Other functional properties of these LDN have not yet been reported.

In viral infections, the presence of LDN has also been reported. In HIV‐infected patients, higher levels of arginase that correlate with lower CD4^+^ T‐cell counts have been reported [149]. Consequently, arginase‐producing cells were found among the PBMC of HIV‐infected patients. These LDN also had a similar morphology to neutrophils, indicating their mature state, and their numbers correlated with disease severity [39]. Another more recent report also confirmed the presence of LDN in HIV‐positive individuals. In addition, these LDN were reported to have increased NET formation [40]. Similarly, in people infected with the virus SARS‐CoV‐2, LDN have been found [41–45]. The number of LDN in circulating blood correlated with COVID‐19 severity [42, 43]. These LDN were reported to have enhanced NET formation [43] and immunosuppressive activity on T cells [41, 44]. Hence, LDN in COVID‐19 may contribute to disease severity by releasing more NET and by inhibiting T‐cell responses.

### 7.4. Pregnancy (Table 2)

In the previous sections, we have discussed how LDN increase in numbers in multiple pathological conditions and that large LDN numbers associate with worse disease outcomes. However, some studies suggest that elevated LDN may also be beneficial. During pregnancy, immune tolerance toward the semi‐allogeneic fetus is essential for a successful gestation. Accordingly, in pregnant women, elevated numbers of LDN have been reported [50, 83, 106, 107]. These LDN (also called G‐MDSC) expressed the enzyme arginase, had enhanced ROS production, and inhibited T‐cell proliferation [107]. LDN included both immature and activated mature neutrophils, both of which seem to increase as pregnancy progresses [50]. After giving birth, the number of LDN decreased within a few days [107]. In comparison, pregnant women who experienced spontaneous abortion did not have an increase of LDN numbers during pregnancy. Also, their LDN presented signs of insufficient activation, as characterized by reduced CD11b expression [83]. In combination, these reports show that LDN increase in normal human pregnancy and imply that LDN may participate in maternal tolerance to the developing fetus.

Moreover, in neonates, LDN are also elevated [51, 106, 108–111, 150]. More LDN are found in cord blood than in blood of children and adults. Cord blood LDN (named G‐MDSC) expressed higher levels of arginase than maternal LDN [106] and suppressed T‐cell functions [109, 111]. These neonate LDN are able to, directly or indirectly, suppress T‐cell responses. Directly, in a contact‐dependent manner with IFN‐*γ* production [150]; and indirectly, by upregulating co‐inhibitory molecules and by downregulating MHC class II molecules on monocytes [110]. LDN augment in cord blood and persist elevated in blood of neonates all through the first month of life. After this time, they decrease to the low levels found in adults [108, 150]. However, if perinatal infections develop, LDN remain longer, and their numbers correlate with indicators of inflammation such as elevated total white blood cell counts and C‐reactive protein levels [108]. Functionally, neonate LDN seem to be defective in phagocytosis and generation of NET [151], and also contribute to impaired phagocytosis of bacteria by monocytes [110, 152]. Further research is required to characterize other antimicrobial functions of LDN in neonates. Together, these studies indicate that elevated LDN during pregnancy may promote maternal tolerance to paternal antigens and contribute to a fruitful pregnancy [153]. However, in newborn babies, although LDN may protect them from inflammatory responses during bacterial commensal colonization after birth, LDN activity may also reduce protective neonate responses to pathogens and promote susceptibility to infection [153].

## 8. Conclusion

LDN are cells with neutrophil morphology and phenotype that are found among the PBMC layer of leukocytes after density gradient centrifugation. The discovery of LDN in SLE revolutionized concepts on the role of neutrophils in disease. Very few LDN are present in the blood of healthy individuals. But LDN numbers increase dramatically in several diseases. In SLE and several inflammatory and infection conditions, LDN display a pro‐inflammatory phenotype, activating immune responses [16, 25, 26, 98, 154]. While in cancer and pregnancy, they perform an immunosuppressive role [57, 84, 153] (as shown in Figure 3). Hence, the particular functional properties of LDN vary depending on the physiological (disease) context they are in. This agrees with the new concept of neutrophil plasticity, resulting in multiple functional phenotypes [10, 14, 15, 134].

The origin of LDN remains a mystery, and therefore, the physical reasons as to why LDN fractionates within PBMC are an important area of investigation. Maturation state and degranulation have been proposed as possible mechanisms for the change in neutrophil density. But, as we have discussed, none of them completely explains the origin of LDN. Another possible mechanism for LDN generation might be the influx of water mediated by aquaporin‐9 [155]. This one and other mechanisms involving activation and functional changes need to be further explored in the future. In addition, it is still possible that LDN derive from a separate neutrophil lineage generated by genomic alterations [66]. Therefore, it remains unclear whether LDN are an extension of a continuous spectrum of activated neutrophils in response to disease‐specific inflammatory cues, or an abnormally developed neutrophil subset distinct from normal neutrophils (as shown is Figure 2).

Although it is now clear that LDN increase in many pathological conditions, including chronic inflammation and infections, there is very little information on the functional properties of LDN in each disease (except maybe SLE). Describing LDN after density centrifugation in a particular illness is important for understanding the role of these cells in each pathology. Characterization of LDN is commonly based on their membrane protein expression, which is detected by flow cytometry. This is a convenient and relatively simple method. However, the tendency to assign a particular cellular function to a certain phenotype should be discouraged, because a phenotype described by flow cytometry analysis is not enough to establish the maturation state or cellular function. Therefore, after a particular phenotype has been determined, LDN should always be purified either by cell sorting or by magnetic selection, in order to evaluate their properties with individual functional assays.

Increasing numbers of LDN seem to correlate with the gravity of various pathologies, for example, asthma, augmented systemic inflammation, trauma, and bacterial infections [31, 33, 41, 105, 147, 148]. In all these cases, LDN numbers might be a good biomarker for disease severity. The same may be true for many other diseases, but this needs to be confirmed by future research. Contrary to the previous examples, elevated numbers of LDN during pregnancy seem to be beneficial, as indicated by pregnant women who did not have an increase of LDN numbers during pregnancy and experienced spontaneous abortion [83]. Consequently, LDN numbers may also be a good biomarker for a successful gestation.

The possible mechanisms by which LDN may influence a particular pathology have only been briefly investigated in a few diseases. In psoriasis [29], arthritis [28], AAV [37], and COVID‐19 [45], LDN are reported to produce more NET and, in this way, promote inflammation and aggravate disease. Similarly, LDN elevated after surgical stress can produce more NET, which, by trapping cancer cells, may promote metastasis [82]. So, disruption of NET with DNase might be a potential way to prevent recurrence after surgery. Together, these reports suggest that NET produced by LDN might be a relevant therapeutic target in several diseases. Whether NET produced by LDN are involved in other pathologies remains to be investigated.

Finally, LDN are a group of neutrophils with unique physical properties and an array of multiple phenotypes and functions. They increase in numerous pathologies, but their particular characteristics in each disease remain largely unexplored. Future research in this field will require to include functional analysis of purified LDN. For this, it is important to established standardized methods in order to be able to compare the properties of LDN in different conditions [116, 156]. In general, LDN, within PBMC, should be identified with at least the following six membrane markers: CD10, CD11b, CD14, CD15, CD16, and C66b. Next, LDN must be isolated (by cell sorting or magnetic columns) and then characterized microscopically and by individual functional assays. The workflow proposed above should also provide enough characteristics of LDN to allow their description within the recently proposed classification system for neutrophils [115]. These strategies will certainly contribute to elucidate the roles of LDN in health and in different diseases.

## Conflicts of Interest

The authors declare no conflicts of interest.

## Author Contributions

Carlos Blanco‐Camarillo suggested the review, collected references, drafted the first manuscript, and prepared figures. Carlos Rosales organized the references, analyzed information, and wrote the article.

## Funding

This research was supported by Grant PAPIIT IN205523 from Dirección General de Asuntos del Personal Académico, Universidad Nacional Autónoma de México (UNAM), Mexico and by Grant CF‐2023‐I‐610 from Secretaría de Ciencia, Humanidades, Tecnología e Innovación (Secihti), Mexico.

## Data Availability

Data sharing is not applicable to this article, as no datasets were generated or analyzed during the current study. The publications that support the findings of this review are available in the References section of this article.
